# Patient-specific finite element analysis of viscoelastic masticatory mucosa

**DOI:** 10.1177/1758736013483298

**Published:** 2013-04-04

**Authors:** Noriyuki Wakabayashi, Tetsuya Suzuki

**Affiliations:** 1 Department of Removable Partial Prosthodontics, Graduate School, Tokyo Medical and Dental University, Tokyo, Japan; 2 Oral Prosthetic Engineering, School of Oral Health Care Sciences, Faculty of Dentistry, Tokyo Medical and Dental University, Tokyo, Japan

**Keywords:** Mucosa, viscoelastic, edentulous, denture, finite element, pain

## Abstract

The purpose of this study was to analyze the stress and strain inside of the oral mucosa in partially-edentulous patients. The patient-specific finite element models of the mucosa and the bone were constructed using the CT images and in-vivo surface measurement during a continuous load. The mean initial shear modulus of 8.3 × 10^–5^ (GPa) and the mean relaxation time of 503 (s) were determined as the viscoelastic properties of the mucosa. The increase of the highest maximum compressive strain during the continuous loading was observed in all the patients, however; the intensity of strain was not in accordance with the thickness of the mucosa. It is suggested that the variations of the morphology and the initial modulus of the mucosa should be considered in the mathematical approaches to detect the mechanical responses of the oral mucosa.

## Introduction

When wearing either conventional or implant-supported removable prostheses, the oral mucosa becomes a significant source of the masticatory force and performs functions that are normally undertaken by the periodontal structures when natural teeth exist. The mucosa prevents excessive occlusal forces from reaching the underlying bone where traumatic resorption can occur in conjunction with other systemic effects.^1,2^ More often, denture wearers complain of pain and damage of the soft tissues under the denture base during mastication.^3^ To prevent the complications of the masticatory mucosa, optimization of denture design and selection of biomaterials should be considered on the basis of the dynamic behavior of the mucosa.

The viscoelastic displacement of the surface of the mucosa was measured and reported in previous animal^4^ and human^5^ studies. However, the stress and strain inside of the mucosa that are directly related to the tissue damage have not been sufficiently evaluated. The soft tissue was rapidly compressed with relatively light force while light loads for long durations deform the tissues more than heavy loads for short durations. This nonlinear response often makes the calculation of the stress and strain a tremendously complex process. In a previous study,^6^ a nonlinear finite element (FE) model of the masticatory mucosa was developed based on the load–displacement relationship of the surface of the mucosa, and *all five subjects in that study* demonstrated the viscoelastic characteristics of the strain intensity inside of the mucosa. However, the influence of variations in the viscoelastic property on the stress and strain of the soft tissues among individual patients has not been assessed.

The number of FE studies that aim to evaluate the mechanical response of the oral mucosa has increased.^7–9^ The stress and strain are mathematical scales that can potentially estimate the pain and damage of the tissues as well as the failures of materials. Such predictions are not possible with the traditional load–displacement data at the tissue surface because the load required for displacement varies greatly depending on the morphology of the tissues and the loading system, while the stress and strain are *scales* independent of such factors. The predictive power by FE analysis is also increased with estimation of the stress/strain *inside* the tissues. However, the variations of the morphology and resilience of the mucosa in individual patients were rarely considered. In the current study, the stress and strain distributions *inside the masticatory mucosa* were analyzed by means of the subject-specific FE models. The purpose of this study was to evaluate the fundamental viscoelastic characteristics in terms of stress and strain inside of the oral mucosa on an individual patient basis. The result can serve as a foundation to assess the influences of location, morphology, and resilience of the denture-supporting mucosa on the stress and strain that could potentially predict pain and damage of the soft tissues.

## Material and methods

**Figure 1.**
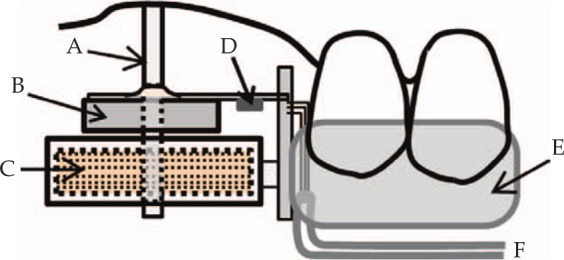
The intraoral measuring device for mucosal displacement. A: loading rod; B: permanent magnet; C: electromagnet; D: strain gauge; E: occlusal appliance; F: cords to amplifier.

The intraoral device consisted of load and sensor components. The loading part included electro- and permanent magnets that were capable of actuating the acrylic rod (1.3 mm in diameter), and the sensor component included a strain gauge (KFC-1-D16; Kyowa electric, Tokyo, Japan) that was attached to a 0.15-mm-thick lead plate (Figure 1). The electromagnet had a resistance of 1.9 kΩ and a driving voltage of 15 V; two permanent 0.35-T magnets were used. The loading rod was connected with the electromagnet on the base of the device and passed through the central cavities of the permanent magnets. Movement of the rod generated strain on the plate, and the signals from the gauge were transferred to the strain amplifier via the control panel outside the mouth. The loading precision was calibrated by fixing the device on a three-dimensional locating stage. The preset loads and the load output from the load cell averaged over eight measurements showed good correlation (*r*^2^ = 0.9963) between 0.05 and 0.50 N.

Three subjects with a loss of maxillary molar teeth on one side (Subjects A, B, and C; mean age = 58.3 years; range = 58–62 years) were recruited from male patients undergoing postoperative maintenance and oral hygiene instructions and had no complaints of pain or soreness. The inclusion criteria were retention of functionally normal dentition mesial to the missing posterior region where the missing teeth had been replaced with a maxillary removable partial denture for at least 3 years. The masticatory mucosa at the top of the residual ridge of the missing maxillary first molar region was analyzed in this study. All experimental procedures were approved by the institutional research ethics committee (No. 01092), and written informed consent was obtained from each subject prior to participation in the study.

An autopolymerized acrylic appliance that covered the occlusal surfaces of the dentition was fabricated. The measuring device was connected to the distal end of the appliance, so that the loading rod was placed on the top of the residual ridge of the missing maxillary first molar region perpendicular to the ridge surface. This occlusal appliance was cemented to the occlusal surfaces of the dentition. The vertical position of the rod was adjusted such that it passively touched the mucosal surface without stress. Under this condition, the force and displacement sensor was reset to zero.

A constant load of 0.05 N was directed onto the mucosal surface and maintained for 10 s, followed by an unloading period of 20 s. This *load creates* an initial pressure of 37,600 Pa on the circular area of the loading rod. The vertical displacement of the rod was measured as the surface displacement during the loading and unloading periods. The measurement was conducted only once a day for each subject to avoid the potential influence of incomplete recovery of the mucosa.

**Figure 2.**
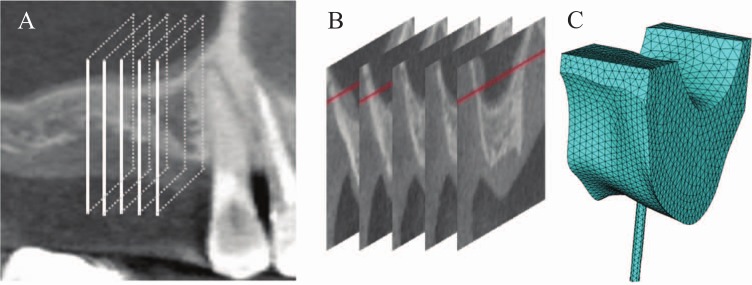
The construction process of a subject-specific model. A: the computed tomography of the maxillary right molar region; B: five buccolingual section images to create a solid volume; C: meshed three-dimensional FE model with the loading rod. FE: finite element

A segmented FE model of the mucosa and the underlying cortical bone in the maxillary first molar vicinity was constructed for each subject on the basis of the digital images derived from cone-beam computed tomography. The axial scan was set at a slice thickness of 0.4 mm with 0.2-mm intervals at 90 kVp and 4.5 mA for 17.5 s. The thickness of the mucosa at the loading site was 3.43, 3.02, and 2.11 mm in subjects A, B, and C, respectively, based on measurements using a multiplanner image reformation. Five buccolingual section images were retrieved between the sites 7 and 17 mm distal to the second premolar at 2.5-mm intervals (Figure 2). Each section was used to form the two-dimensional outline, and the outlines of all the sections were connected and posteriorly extended to create a three-dimensional solid volume. The base of each bone model was created as a horizontal plane close to the maxillary sinus, so that the vertical height of the entire model was 25 mm. Each bone volume was divided into the cortical and cancellous core, and the model base was created with the cancellous bone at the horizontal level corresponding to the palatal vault. The acrylic loading rod was also modeled as a cylinder with identical dimensions. Each model was meshed by approximately 35,000 nodes and 21,000 elements defined by 20 nodes having 3 degrees of freedom. The surface-to-surface contact elements were applied to the loading plane of the rod and the mucosal surface with a coefficient of friction of 0.25. The mesh size and arrangement were determined on the basis of the preliminary convergence tests for consistency of the solutions.^10^ The cortical and cancellous structures were assumed to be homogeneous, isotropic, and linearly elastic. Young’s modulus was 14, 0.5, and 9 GPa for the cortical bone, cancellous bone, and acrylic pin, respectively, with a Poisson’s ratio of 0.3 for all structures.^11–13^

The mucosa was also assumed to be homogeneous, isotropic, and *fixed* to the cortical bone. The initial shear modulus (G_0_) and the relaxation time (τ) for the nonlinear phases of each subject were determined according to the procedures described in the previous study.^6^ Each model with determined G_0_ and τ values was used to calculate the distribution of stress and strain within the small segmented soft tissues of each subject. The boundary condition included fixation of the upper base of each model in addition to symmetrical restriction of the mesial and distal surfaces. The model accuracy was verified by reproducibility of the same model used in an additional experiment with larger loads up to 0.1 N. For all calculations, the magnitude and directions of the principal stress and strain of the mucosa were analyzed. The stress and strain were plotted along the vertical central axis of the loading rod. The center of the mucosa was defined as the spot at a depth half the original thickness of each mucosa before loading. Because the compressive state was dominantly shown in the oral mucosa of this study, the third (minimum) principal stress and strain values were calculated as the maximum compressive stress and strain.

## Results

**Figure 3.**
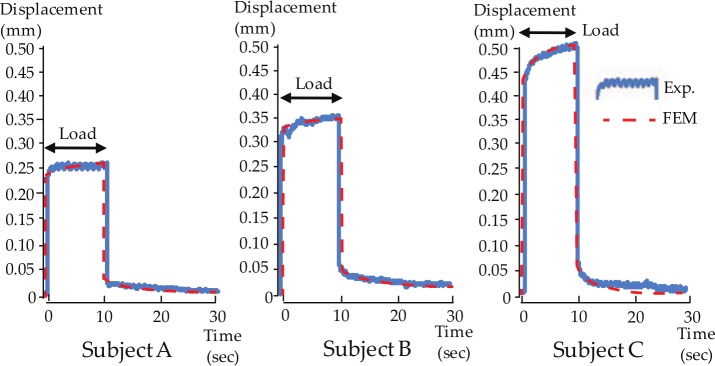
Surface displacement of the mucosa under continuous loading. The experimental time-displacement relationship is indicated by a solid curve, and the curve adaptation of the FE analysis is shown by a dotted curve.

In the in vivo experiment, the surface displacement of the mucosa of all subjects showed an instantaneous increase in the vertical displacement upon loading, followed by an increase of the intrusion during the continuous loading (blue curve, Figure 3). Upon unloading, instantaneous recovery occurred, followed by delayed gradual recovery. The percentage of the displacement recovery immediately after unloading relative to the maximum displacement after 10-s continuous load was 87.3% on average, increasing to 97.7% recovery after 20-s unloading (n = 3). The G_0_ of 9.5, 8.5, and 7.0 (10^−5^ GPa) were determined for the subjects A, B, and C, respectively (mean = 8.3 × 10^−5^ GPa). The mean ± standard deviation of τ was 503 ± 46 s (Table 1).

**Table 1.** Vertical surface displacement of the mucosa in the experiment. The loading of 10 s followed by an unloading of 20 s was performed for each patient.

	Immediately after loading (mm)	After continuous loading (mm)	Immediately after unloading (mm)	20 s unloading (mm)	Increase rate by continuous load (%)	Immediate recovery rate (%)	20 s recovery rate (%)	G_0_ (×10^−5^ GPa)	τ (s)
Time (s)	A (0.1)	B (9.9)	C (10.1)	D (30.0)	B/A	(B−C)/B	(B−D)/B		
Patient A	0.24	0.24	0.02	0.00	101.2	90.9	98.9	9.5	510
Patient B	0.39	0.42	0.06	0.02	108.3	85.0	95.0	8.5	487
Patient C	0.47	0.53	0.07	0.00	113.9	86.1	99.2	7.0	512
Average	0.36	0.40	0.05	0.01	107.8	87.3	97.7	8.3	503
SD	0.22	0.27	0.06	0.02	11.4	7.2	5.1	2.0	46

SD: standard deviation.

**Figure 4.**
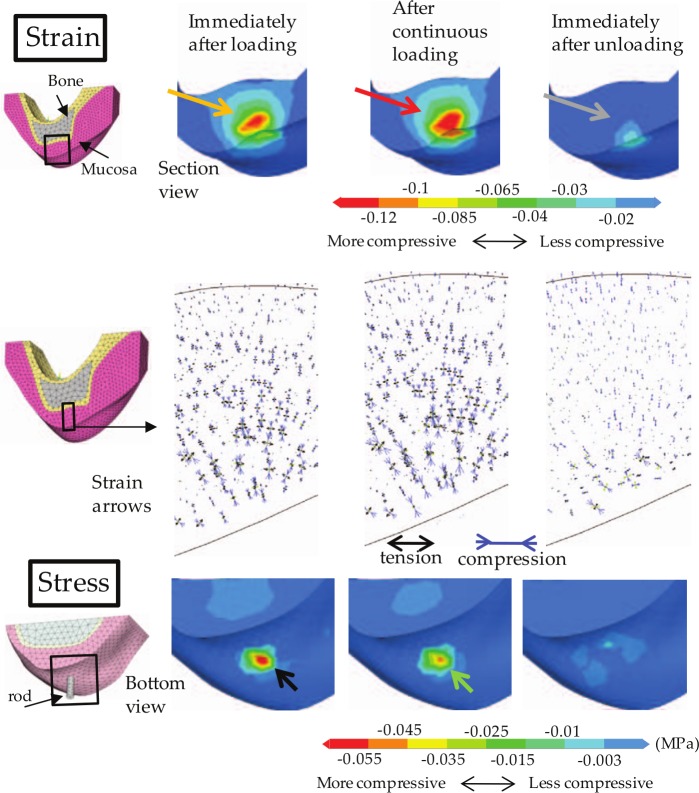
The strain and stress distributions. Left figures indicate the regions of interest by black boxes. The graphics indicate, from left to right, the contours at the time immediately after onset of loading, after the 10-s continuous loading, and at the time immediately after the unloading. The contours on the upper row represent the maximum compressive strain. The arrow graphics on the middle row show the strain directions. The contours on the bottom indicate the maximum compressive stress.

The strain and stress distributions are shown by the contour graphics of subject B (Figure 4). The highest maximum strain immediately after onset of loading was observed in the subsurface region under the edge of the loading rod (orange arrow). The area of the highest compression increased with the continuous load (red arrow). The residual strain was present immediately after unloading (gray arrow). The maximum compressive *stress* was observed immediately after loading (black arrow) but it was followed by a decrease of the highest stress area after the continuous load (light green arrow).

**Figure 5.**
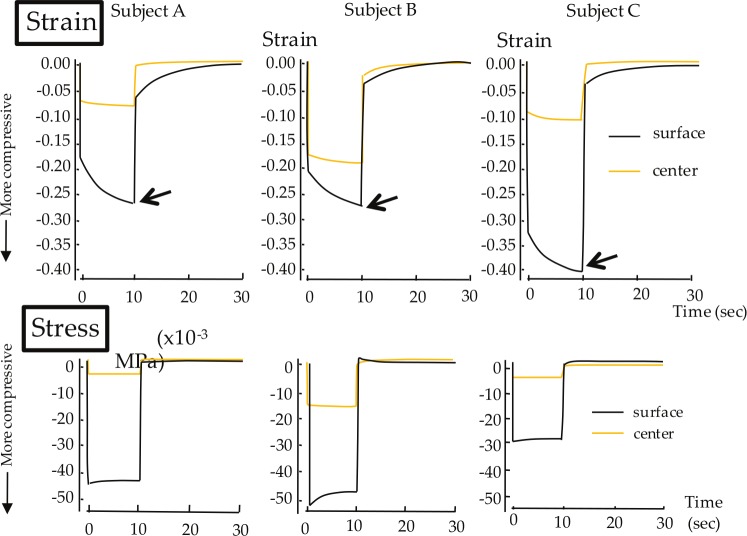
The strain and stress as a function of time. The minimum third principal (compressive) strain and stress at the surface and center of the mucosa throughout the loading and unloading periods for each subject.

The maximum compressive strain and stress at the surface and the center of the mucosa right under the central axis of the loading rod were graphed as a function of time for each subject (Figure 5). It should be noticed that lower minimum principal strain and stress in the negative scale represent higher compression. At the surface, the highest maximum compressive strain was recorded after a continuous period for all subjects (black arrows). The maximum strain was more compressive at the surface, with only a minor increase (downward curve) over the loading period. In subject B, the strain at the center reached 67% of that at the surface, while it was less than 30% in the other subjects. The compressive *stress* that was generated at the surface slightly decreased during loading in subjects A and C, while it markedly decreased in subject B. At the center, no detectable change was found with respect to the *stress* over the loading period.

## Discussion

The surface displacement of masticatory mucosa of all subjects exhibited typical viscoelastic behavior similar to the finding of the previous study (Figure 3).^14^ On the contrary, the vertical intrusion of the masticatory mucosa was not in accordance with the thickness of the mucosa. Indeed, subject A, who had the thickest mucosa, exhibited the lowest maximum displacement (Figure 3), while subject C, who had a thin mucosa, exhibited the highest displacement probably because of the lowest modulus (G_0_). A low modulus is indicative of flexible characteristics that usually cause the likelihood of a large deformation under loading. Although the subject C showed the greatest maximum compressive strain in the surface region, the compressive strain inside of the mucosa was less than that of subject B of higher modulus. In subject C with a thin mucosa, the compressive strain at the center of mucosa was suppressed presumably because of the proximity of the rigid cortical bone. Within the limitations of the study design using the segmented small mucosal volumes of three subjects, the result of this study suggests that the initial modulus as a part of the viscoelastic property is one of the determinant factors of the strain magnitude inside of the soft tissues. Further studies are encouraged to establish the rigidity-dependent viscoelastic properties with the loading by the actual denture base, which would facilitate optimal design of prostheses.

The deformation of mucosa is largely a function of fluid interchange with surrounding unstressed mucoperiosteum. The viscoelastic characteristics are attributed to the displacement of blood and tissue elements as well as the distortion of large polymer molecules of the soft connective tissues.^15^ In the models of this study, the maximum strain under the continuous load increased at the region near the surface (Figure 5, strain). The gradual distortion that occurred in the connective tissues indicated that the morphology of the denture-supporting mucosa changes with chewing.^16^ It is suggested that duration of the load is a critical factor that causes strain increment near the surface. On the contrary, the maximum strain at the center of mucosa was relatively constant. The inner mucosa exhibits distinct resistance to deformation under an applied load because of its firm attachment to rigid cortical bone, while the nonrigid surface of the mucosa, without firm support from the bone, exhibits viscous behavior during sustained loading.^17^ Meanwhile, the mathematical models, in which the soft tissue is depicted as a homogeneous structure, should be considered in further detail. Inclusion of roles of each tissue component in the models might affect the stress and strain values to some degree. However, the difference in rigidity between tissue components must be considerably smaller than that between the soft tissues and cortical bone, and the model depicted the bottom of the mucosa firmly attached to rigid cortical bone, which is the most influential aspect of the mechanical characteristics of these tissues. Therefore, these facts played more significant roles in the results than the assumed homogeneous structure. However, a structural model consisting of tissue layers with different mechanical properties,^18^ such as vessels, collagen bundles, and connective tissues, should be developed to account for the roles of each layer in accommodating viscoelasticity.

The maximum compressive stress decreased slightly during the loading period in all subjects (Figures 4 and 5, stress), indicating that the delayed increase in strain is not accompanied by a simultaneous increase in stress. During stress relaxation or a gradual decrease of the maximum compressive stress under constant displacement at the surface, the high intensity of stress might be relieved and dispersed to a wider region of the mucosa. It is hypothesized that the viscoelastic behavior plays a self-defensive role in relieving stress concentration that can cause tissue damage or degradation. The stress and strain of the oral mucosa were regulated in relation to the viscoelastic property of the soft tissue, and this varied among patients. The result can potentially explain the mechanism and region-specific modulation of the pressure–pain threshold of the human oral mucosa.^19^ For optimization of the prosthodontic design, extensive mechanobiological research is encouraged to investigate the ultimate strain threshold that may cause the soft tissue damage.^20^

The use of the small segmented model was a reasonable approach to explore the general characteristics of the mechanical response of oral mucosa. Measurement using a large denture base of a curved surface would result in unequal displacements at different sites, and therefore would not be useful as a general model. In this study, the stress and strain data were plotted under the central axis of the loading rod, eliminating the result of the peripheral areas that could exhibit the edge effect. The magnitudes of strain were relatively small because of the loading regimen that offered an average pressure imposed on a denture-bearing mucosa. However, the potential risk for damage should not be underestimated because the stress could dramatically increase if a thin mucosa is attached to sharp-edged or irregular-shaped bone.^21^ In this study, the fundamental viscoelastic characteristics were analyzed and assessed in terms of stress and strain on an individual patient basis. The result suggests that the difference in the mechanical properties of the masticatory mucosa between subjects can affect the viscoelastic characteristics inside of the mucosa. The variations of the morphology as well as the rigidity of the mucosa should be considered in future mathematical approaches aiming to assess the responses of the masticatory mucosa.

## Conclusion

The subject-specific FE method with loading experiment demonstrated that the surface displacement and the strain intensity during the continuous load exhibited typical viscoelastic behavior in the masticatory mucosa of the partially edentulous patients. The mean initial shear modulus of 8.3 × 10^−5^ GPa and the mean relaxation time of 503 s were determined as the viscoelastic properties of the mucosa. The increase of the highest maximum compressive strain during the continuous loading was observed at the region near the loading surface while the intensity of the strain differed considerably among subjects probably due to variations of thickness and modulus. It is suggested that the viscoelastic property of individual patient should be considered in future mathematical approaches to detect the mechanical responses of the soft tissues.
